# Short-term intensive fasting enhances the immune function of red blood cells in humans

**DOI:** 10.1186/s12979-023-00359-3

**Published:** 2023-08-30

**Authors:** Yixuan Fang, Jiawei Qian, Li Xu, Wen Wei, Wenwen Bu, Suping Zhang, Yaqi Lv, Lei Li, Chen Zhao, Xueqin Gao, Yue Gu, Li Wang, Zixing Chen, Xiao Wang, Ruizhi Zhang, Youjia Xu, Yanjun Yang, Jie Lu, Zhanjun Yan, Mingyuan Wang, Longhai Tang, Na Yuan, Jianrong Wang

**Affiliations:** 1 Research Center for Blood Engineering and Manufacturing, Cyrus Tang Medical Institute, Suzhou Medical College of Soochow University, Suzhou, 215123 China; 2 National Research Center for Hematological Diseases, State Key Laboratory of Radiation Medicine and Protection, Collaborative Innovation Center of Hematology, Soochow University, Soochow, China; 3 Department of Community Nursing, Soochow University, Suzhou, China; 4 Jiangsu Institute of Hematology, The First Affiliated Hospital of Soochow University, Soochow, China; 5 The Second Affiliated Hospital of Soochow University, Soochow, China; 6 The Ninth Affiliated Suzhou Hospital of Soochow University, Soochow, China; 7 Suzhou Blood Center, Suzhou, 215006 China

**Keywords:** Fasting, Red blood cells, Immune response, Infectious disease, *SARS-CoV-2*

## Abstract

**Background:**

Fasting is known to influence the immune functions of leukocytes primarily by regulating their mobilization and redistribution between the bone marrow and the peripheral tissues or circulation, in particular via relocalization of leukocytes back in the bone marrow. However, how the immune system responds to the increased risk of invasion by infectious pathogens with fewer leukocytes in the peripheral blood during fasting intervention remains an open question.

**Results:**

We used proteomic, biochemical and flow cytometric tools to evaluate the impact of short-term intensive fasting (STIF), known as beego, on red blood cells by profiling the cells from the STIF subjects before and after 6 days of fasting and 6 days of gradual refeeding. We found that STIF, by triggering the activation of the complement system via the complement receptor on the membrane of red blood cells, boosts fairly sustainable function of red blood cells in immune responses in close relation to various pathogens, including viruses, bacteria and parasites, particularly with the pronounced capacity to defend against *SARS-CoV-2*, without compromising their oxygen delivery capacity and viability.

**Conclusion:**

STIF fosters the immune function of red blood cells and therefore, it may be considered as a nonmedical intervention option for the stronger capacity of red blood cells to combat infectious diseases.

**Supplementary Information:**

The online version contains supplementary material available at 10.1186/s12979-023-00359-3.

## Keypoints

STIF fosters the immune response of red blood cells to various pathogens, in particular *SARS-CoV-2*, via activation of the complement receptor on their membranes.

STIF does not compromise the oxygen transport capacity or viability of red blood cells.

**Graphical abstract:**

**Supplementary Information:**

The online version contains supplementary material available at 10.1186/s12979-023-00359-3.

## Graphical abstract

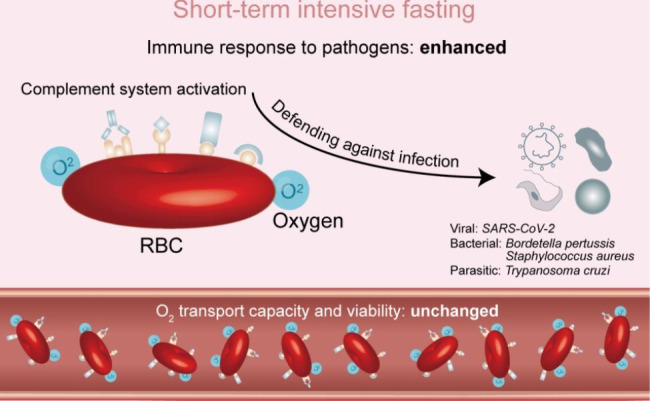

## Introduction

The major function of red blood cells is delivery of oxygen to all tissues across the body; however, immunoregulatory properties of this type of cell have also been documented. Early studies indicated that the immune function of red blood cells appeared to be restricted to their immature counterparts and play largely suppressive roles in newborn mice [1, 2]. For example, CD71^+^ erythroid cells, by expressing arginase-2 and/or the surface molecule V-domain Ig suppressor of T-cell activation (VISTA), have immunosuppressive activity and inhibit both the innate and adaptive immune responses in newborn mice [3–5]. In humans, neonatal CD71^+^ erythroid cells were also found to regulate T-cell and myeloid responses [6].

Unlike the immune suppressive roles of premature red blood cells or red blood cells in newborns, the complement system on mature red blood cells in humans was found to be an important defense mechanism against pathogens. In particular, complement C3b/C4b receptor (CR1, CD35) is capable of binding most complement-fixing immune complexes in the circulation so that red blood cells retain immune complexes on their membranes in the intravascular space and deliver them to the tissue macrophages for clearance, primarily in the liver and spleen [7–10]. As red blood cells traverse the liver, immune complexes are removed from their membranes, releasing the red blood cells unharmed into circulation [8, 10–13]. Mechanistically, after binding to the complexes on the membrane of red blood cells, complement C3b is catabolized by factor I, together with its receptor CR1 as a cofactor [14]. In this way, the complement-activating properties of the complexes are reduced to prevent immune complexes in the vessel walls, securing safe and effective capture and subsequent elimination of immune complexes in human circulation [15]. Using single-cell transcriptomic analysis, the Shi group identified an immune-prone population in erythroid precursors throughout human ontogenesis. These immune-erythroid cells were found to couple with dual erythroid and immune regulatory networks and play immunomodulatory roles by actively interacting with various immune cells, suggesting that red blood cells possess immune function throughout our entire lifespan [16].

Fasting has been reported to impact immune functions in many aspects by redistributing immune cells/leukocytes between the bone marrow and peripheral circulation. For example, T lymphocytes relocate from secondary lymphoid organs to the bone marrow during caloric restriction in mice [17]. Fasting causes B cells to leave Peyer’s patches in the small intestine of mice [18] and reduces the number of circulating monocytes in mice and humans by preventing their mobilization from the bone marrow [19]. During fasting, monocyte re-entry to the bone marrow is orchestrated by hypothalamic‒pituitary‒adrenal axis-dependent release of corticosterone augmenting chemokine receptor 4 (CXCL4) [20]. Our study shows that STIF, a traditional fasting regimen consisting of a water-only fasting period and a gradual refeeding program mainly practiced in China and several Asian countries, remodels the innate immunity of neutrophils in humans [21]. However, it remains largely unclear whether fasting of various formats influences the function of nonleukocyte cells.

To make STIF or beego easier to practice, it combines water-only fasting with a psychological induction component that includes meditation and abdominal breathing, light body exercise, and ends with a gradual refeeding program before returning to a normal diet. Here, we used proteomics, biochemical and cytometric tools to study the impact of STIF on functional changes in red blood cells in humans.

## Results

### STIF promotes the immune function of red blood cells

**Fig. 1:**
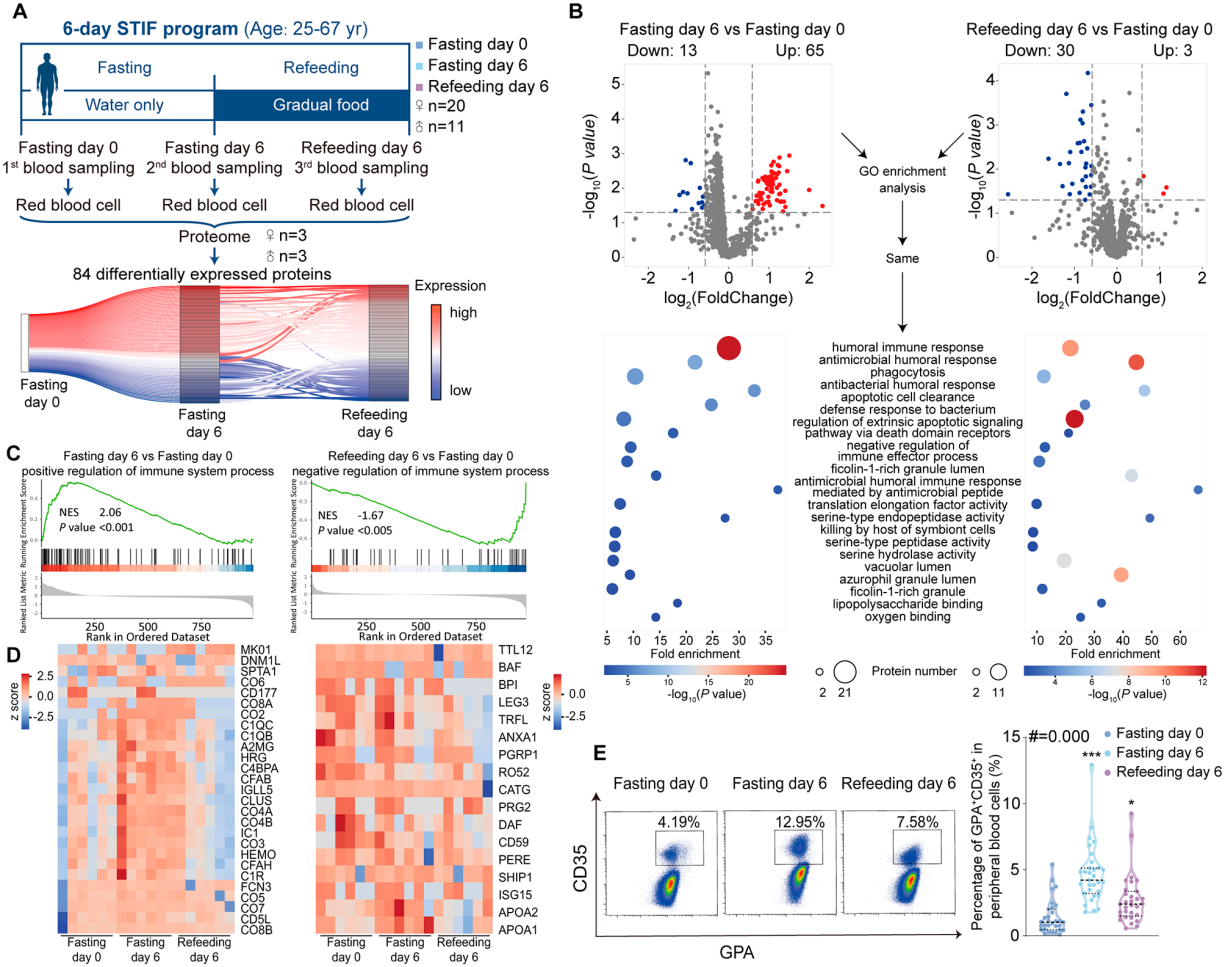
Analysis of overall immune responses of red blood cells to STIF by proteomics and flow cytometry. **(A)** Schematic diagram of the 6-day STIF program and expression dynamics of DEPs. Upper, Schematic illustration of the STIF program that includes fasting for 6 days and gradual refeeding for 6 days (n = 31). Bottom, Expression dynamics of all DEPs between STIF day 0, STIF day 6 and refeeding day 6. Protein abundance at the later time points was normalized to STIF day 0 as relative expression. **(B)** Differential protein expression and GO analysis of red blood cells. Top, Volcano plot of the red blood cell proteome. Lower panel, GO enrichment analysis of the differential proteome. Left, fasting day 6 vs. fasting day 0, right, refeeding day 6 vs. fasting day 0. The color bar indicates -log_10_(*P* value). The bubble size indicates the number of DEPs annotated to a GO term. The horizontal axis is the fold enrichment (gene ratio/background gene ratio) in GO enrichment analysis. The vertical axis is the name of the GO term. **(C)** GSEA on the regulation of immune system processes. **(D)** Annotated heatmap of protein abundances related to positive regulation of immune system process (left) and negative regulation of immune system process (right). **(E)** Flow cytometric quantitation of the immune function of red blood cells (GPA^+^) marked by CD35. Fasting day 0 (dark blue), fasting day 6 (sky blue), refeeding day 6 (purple), n = 31/group

To explore the general impact of STIF on red blood cells, thirty-one subjects performed short-term intensive fasting (STIF) for six days, immediately followed by a gradual refeeding period for another six days, a regimen currently favored by water-only fasting participants in Asian countries (Fig. 1A, upper panel). We acquired proteomics profiles of red blood cells from 6 STIF subjects (three men and three women randomly selected from 31 STIF cohort) providing 18 blood samples to identify the difference in protein abundance before and after STIF/refeeding (Table S1, Figure S1). A total of 1009 proteins were identified in human red blood cells. Differential analysis yielded 84 differentially expressed proteins between the three time points, forming different expression dynamic patterns in the fasting/refeeding process (Fig. 1A, lower panel). Paired analysis produced a total of 78 differentially expressed proteins (DEPs) on fasting day 6, with 65 proteins upregulated and 13 proteins downregulated (Fig. 1B, left). There were still 33 differentially expressed proteins on refeeding day 6, in which 3 proteins were upregulated and 30 proteins were downregulated (Fig. 1B, right). To determine biological pathways that are activated or silenced during the intensive fasting and refeeding process, we employed overrepresentation analysis on the proteome [22]. Pathways with *P* values lower than 0.05 were considered biologically insightful. The enrichment analysis of significant differentially expressed proteins produced 147 statistically meaningful pathways on fasting day 6 and 87 pathways on refeeding day 6 in Gene Ontology (GO). Fasting day 6 and refeeding day 6 shared 20 of the same pathways. Almost all of these pathways are associated with immune responses (Fig. 1B, down). We used gene set enrichment analysis (GSEA) to incorporate the differential expression analysis into the enrichment process to quantify the positive or negative effect of a given pathway. GSEA revealed that compared to fasting day 0, positive regulation of the immune system process was activated and negative regulation of the immune system process was unchanged on fasting day 6; otherwise, negative regulation of the immune system process was inhibited and positive regulation of the immune system process was unchanged on refeeding day 6 (Fig. 1C). Proteins involved in positive regulation of the immune system process were upregulated on fasting day 6, whereas proteins involved in negative regulation of the immune system process were downregulated on refeeding day 6, suggesting that STIF promotes the immune function of red blood cells (Fig. 1D). Flow cytometric analysis of the red blood cells from fasting subjects showed that the expression of CD35 protein (CR1) on red blood cells, a marker known to positively correlate with the enhanced immune response in red blood cells [23], was significantly increased after STIF (Fig. 1E), again suggesting an increased immune function by STIF.

### The immune responses of red blood cells activated by STIF are characterized by the response to viruses, bacteria and parasites, especially *SARS-CoV-2*

**Fig. 2:**
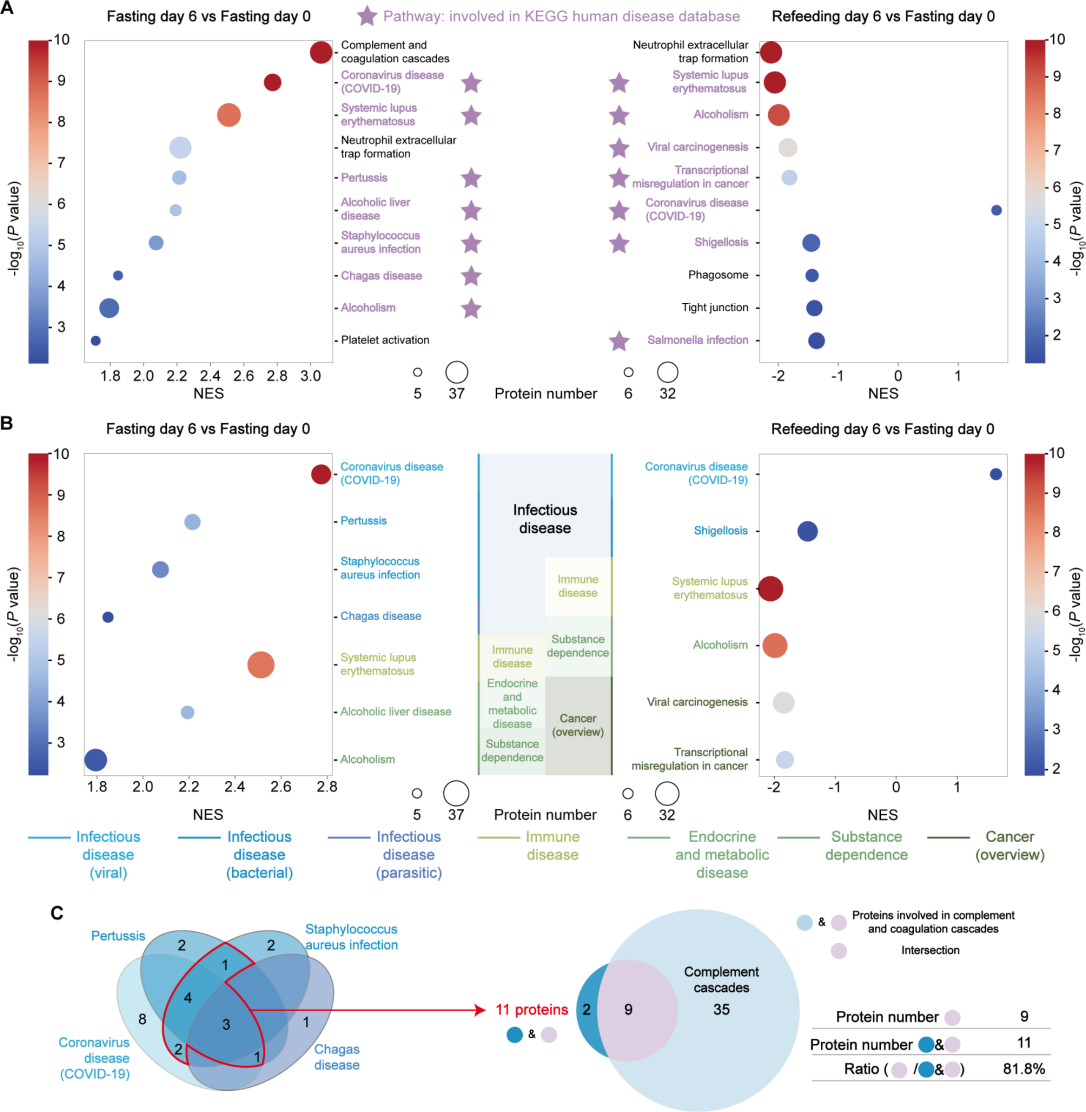
Proteomic profiling of STIF-triggered immune responses of red blood cells to pathogen infection. **(A)** The top ten KEGG pathways enriched in red blood cells by STIF were obtained by GSEA. The pathway marked by the purple pentagram (★) is involved in the KEGG human disease database. The colormap corresponds to -log_10_(*P* value) of GSEA. The bubble size indicates the number of proteins annotated to a KEGG pathway. The horizontal axis is the value of NES (normalized enrichment score) in GSEA. The vertical axis is set as the names of KEGG pathways. **(B)** Pathways involved in the KEGG human disease database were obtained by GSEA, and disease categories are distinguished by color. Infectious diseases are marked in blue, including viral, bacterial and parasitic diseases. Other diseases are marked in green, including immune disease, endocrine and metabolic disease, substance dependence and cancer. Pathways were filtered by *P* value < 0.05 and |NES|≥1. The colormap corresponds to -log_10_(*P* value) of GSEA. The horizontal axis is the value of NES in GSEA. The vertical axis is set as the names of KEGG human disease pathways. **(C)** Venn diagram depicting eleven intersecting proteins in at least two pathways, nine of which are involved in the complement system

To understand the immune functional changes of red blood cells by STIF, we ran GSEA on our proteome profile with the KEGG (Kyoto Encyclopedia of Genes and Genomes) database. We found that seven of the top ten pathways, ranked by *P* value, were the major part of the KEGG human disease database. These seven KEGG human disease pathways, marked by a purple pentagram, are diseases caused by pathogen infection (Fig. 2A). This analysis suggests that STIF mainly affects the red blood cell response to pathogens. Therefore, we paid more attention to the effects of red blood cells responding to pathogens after STIF.

Among the top ten upregulated KEGG pathways on fasting day 6, we observed enhanced immune responses, largely to various pathogens, including viruses, bacteria and parasites that cause respiratory disease by *SARS-CoV-2*, pertussis by *Bordetella pertussis*, staph infection by *Staphylococcus aureus*, and Chagas disease by *Trypanosoma cruzi* (Fig. 2B, left). There were three identical pathways enriched in both fasting day 6 vs. fasting day 0 and refeeding day 6 vs. fasting day 0. They are coronavirus disease (COVID-19), systemic lupus erythematosus and alcoholism. Systemic lupus erythematosus and alcoholism were both upregulated on fasting day 6 and downregulated on refeeding day 6, suggesting that refeeding would quickly offset this effect of fasting. Therefore, STIF activates immune responses to various infectious diseases caused by viruses, bacteria or parasites but has a lasting effect solely on coronavirus disease (COVID-19) (Fig. 2B).

To characterize the four upregulated infectious disease pathways, we screened for eleven proteins (marked by red lines) that were detected in at least two of the pathways (Fig. 2C, left). Furthermore, nine of these eleven proteins were found to be involved in the complement system (Fig. 2C, right). This analysis suggests that the upregulation of the four infectious disease pathways is closely related to the change in proteins in the complement system.

### STIF activates the complement system via complement receptors on the membrane of red blood cells and fortifies their capacity to fight against *SARS-CoV-2*

**Fig. 3:**
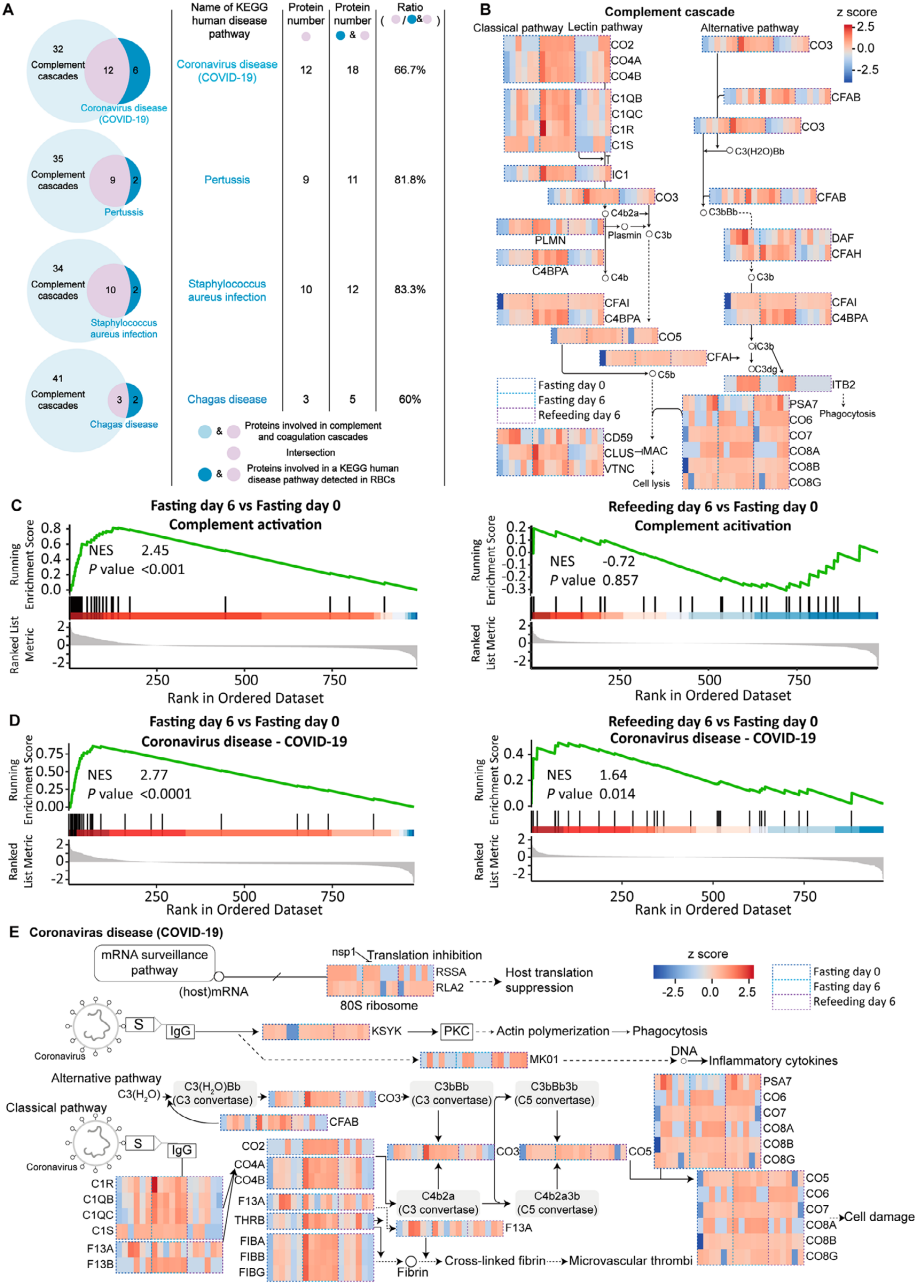
Proteomic identification of the responses of the complement system on the membrane of red blood cells to STIF. **(A)** Two-way Venn diagram comparing proteins involved in the infectious disease pathway detected in red blood cells and proteins in complement cascades. Left, Venn diagram. Right, calculation of the ratio of intersecting protein number between infectious disease pathways and the complement cascade to the detected protein number involved in the infectious disease pathway. **(B)** Annotated heatmap of proteins involved in the complement system. The signaling pathway is adapted from the KEGG pathway hsa04610. **(C)** GSEA on complement activation. **(D)** GSEA on the regulation of coronavirus disease (COVID-19). **(E)** Annotated heatmap of proteins involved in coronavirus disease (COVID-19). The signaling pathway is adapted from the KEGG pathway hsa05171

To briefly determine the possible mechanism responsible for the upregulation of immune responses against pathogens identified above (Fig. 2), we performed Venn diagram analysis, which compared proteins involved in four infectious diseases upregulated on fasting day 6 and proteins involved in complement cascades (Fig. 3A, left). The number of overlapping proteins in the Venn diagram accounts for more than 60% of the number of proteins involved in the infectious disease pathway. More than 80% of the proteins in two of the infectious disease pathways are proteins in the complement system (Fig. 3A, right). This suggests that the upregulation of these four infectious disease pathways is mainly linked to complement activation.

The complement system on mature red blood cells is an important defense mechanism against pathogens[11, 23]. In accordance with the results from Venn diagram analysis, profiling of DEPs proposed that the proteins involved in positive regulation of the immune system process are mainly related to complement cascades (Fig. 1D, left). The expression dynamics and GSEA on complement cascades further confirmed that STIF activates the complement system (Fig. 3B, C), thereby suggesting that activation of complement is the main cascade by which STIF promotes erythrocyte immune function.

Intriguingly, GSEA revealed that red blood cells were upregulated in the biological processes relevant to fighting against *SARS-CoV-2* after STIF (Fig. 3D). Specifically, STIF activates the complement system, with both classical and alternative pathways upregulated, which are involved in the coronavirus disease (COVID-19) KEGG pathway (Fig. 3E), reminiscent of previous studies showing that complements are activated on the erythrocytes of patients with COVID-19[24–26].

### STIF maintains the oxygen transport function of red blood cells

**Fig. 4:**
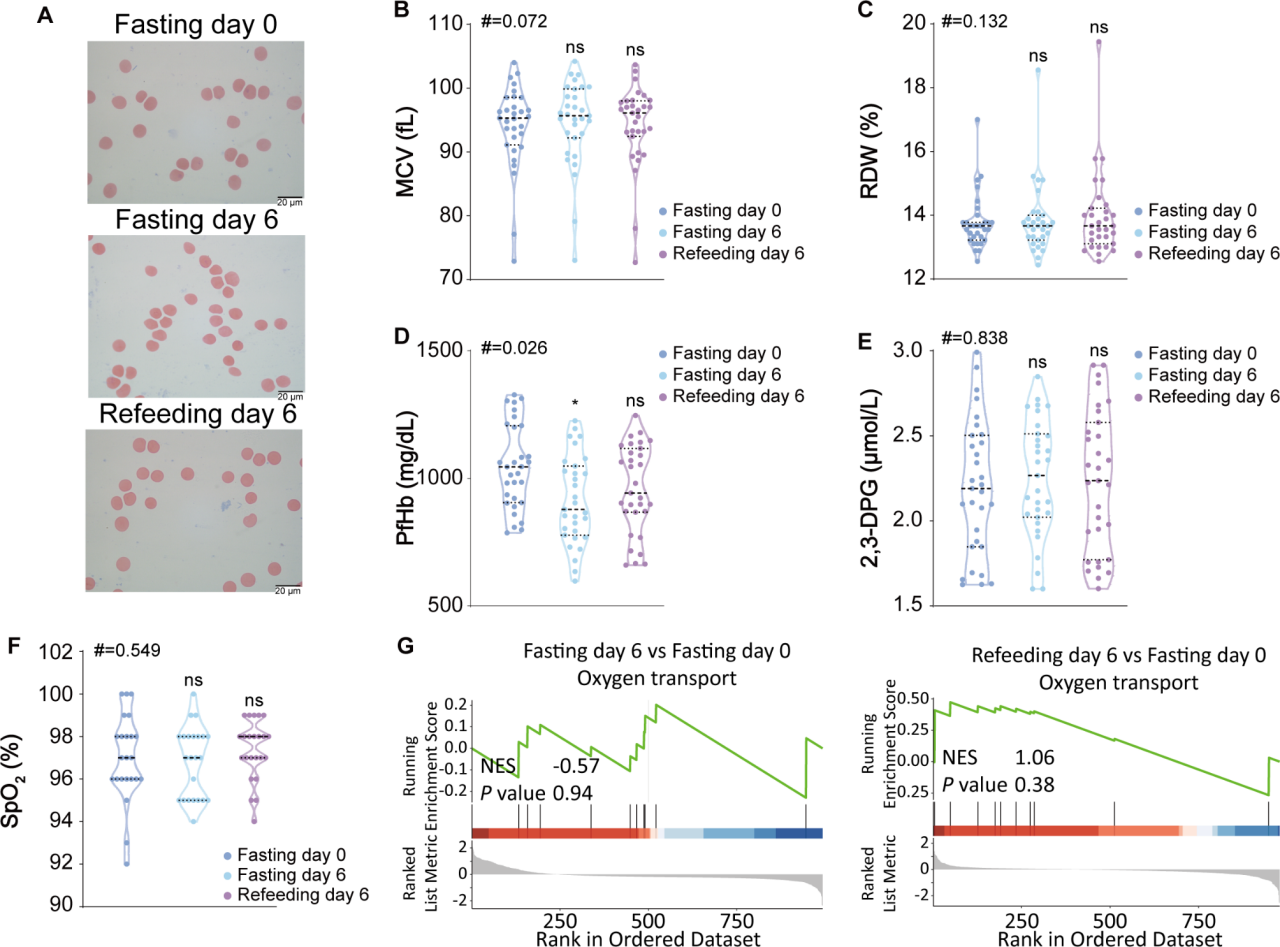
Biochemical and proteomic examination of the effect of STIF on oxygen transport function in red blood cells. **(A)** Peripheral blood smear by Wright-Giemsa stain. **(B**) Mean corpuscular volume (MCV) test (n = 31). **(C)** Red cell distribution width (RDW) test (n = 31). **(D)** Plasma free hemoglobin (PfHb) measured by ELISA (n = 31). **(E) **2,3-Diphosphoglycerate (2,3-DPG) concentration of peripheral red blood cells measured by ELISA (n = 31). **(F)** Oxygen saturation determined by a finger clip blood oxygen analyzer (n = 23). **(G)** GSEA on oxygen transport

To examine whether STIF enhances capacities in the immune response at the cost of the main function of red blood cells in oxygen transport, we performed the following laboratory tests that could define the function of red blood cells. First, blood smears did not show noticeable changes in morphology (Fig. 4A), nor did the average size of red blood cells, represented by the mean corpuscular volume (MCV) (Fig. 4B), or the range in the volume and size of red blood cells, represented by the red blood cell distribution width (RDW) change (Fig. 4C). Second, we measured the level of plasma free hemoglobin (PfHb) in the serum of the STIF subjects since PfHb causes excessive red blood cell breakdown and induces vasoconstriction; thus, increasing PfHb levels are associated with increased vasoconstriction but decreased microvascular density [27]. The results showed a decreased level of PfHb release after STIF (Fig. 4D), suggesting that red blood cells had a reduced risk of damaging endothelial function after STIF. Next, we measured the 2,3-diphosphoglycerate (2,3-DPG) level, which reflects the ability of red blood cells to regulate oxygen binding to HGB [28]. STIF did not alter the level of 2,3-DPG (Fig. 4E), suggesting no change in oxygen-carrying function per HGB protein. Furthermore, we performed the SpO_2_ assay, which directly indicates the level of oxygenated HGB in red blood cells, and the results showed that STIF did not change the capacity of HGB to bind to oxygen in red blood cells (Fig. 4F), reflecting the unperturbed oxygen transportation capacity per HGB molecule in the body. In addition to the above biological assays, GSEA revealed that compared to fasting day 0, proteins involved in oxygen transport were not changed on fasting day 6 and refeeding day 6 (Fig. 4G). These results together suggest that STIF maintains the oxygen transport function of red blood cells.

### STIF maintains the viability of red blood cells

**Fig. 5:**
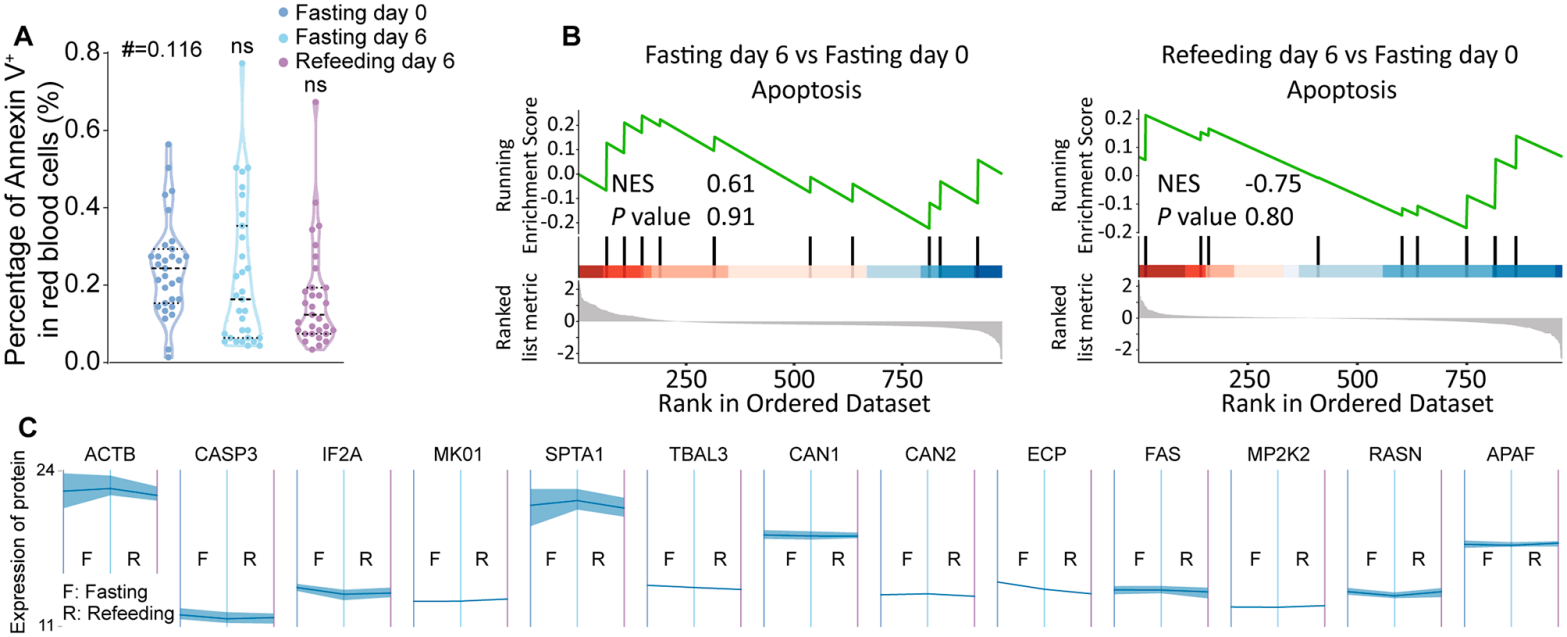
Biochemical and proteomic examination of the effect of STIF on the survival of red blood cells. **(A)** Percentage of apoptosis (Annexin V^+^ cells) in red blood cells. **(B)** GSEA on apoptosis. **(C)** Time series analysis of apoptosis-related proteins in red blood cells. ACTB, actin beta. CASP3, caspase 3. IF2A, eukaryotic translation initiation factor 2 subunit 1. MK01, mitogen-activated protein kinase 1. SPTA1, spectrin alpha chain, erythrocytic (1) TBAL3, tubulin alpha chain-like 3. CAN1, calpain-1 catalytic subunit. CAN2, calpain-2 catalytic subunit. ECP, eosinophil cationic protein. FAS, fas cell surface death receptor. MP2K2, dual specificity mitogen-activated protein kinase kinase (2) RASN, GTPase NRas. APAF, apoptotic protease-activating factor 1

To evaluate the influence of STIF on erythrocyte survival, we tested the percentage of Annexin V^+^ in RBCs and analyzed dynamic changes in apoptosis-related proteins by GSEA and time series analysis. The results showed that there was no change in the percentage of Annexin V-positive cells during STIF (Fig. 5A). Similarly, GSEA revealed that compared to fasting day 0, apoptosis was not changed on fasting day 6 and refeeding day 6 (Fig. 5B). Finally, time series analysis of proteins involved in apoptosis showed no change during STIF (Fig. 5C). Although a previous study reported that overactivation of complement induces caspase activation and apoptosis [29], the unchanged level of apoptosis suggests that complement activation by STIF did not interfere with erythrocyte viability.

## Discussion

In this study, we found that in humans, STIF can boost the function of red blood cells in the immune response to various types of pathogens, particularly in fighting against *SARS-CoV-2*, by activating the complement cascade on their membranes without compromising their oxygen transport capacity and viability. STIF-enhanced immune function is fairly sustainable since such positive effects are maintained not only after 6 days of fasting but also after a 6-day refeeding period.

Previous studies indicate that dietary restriction and fasting of various formats influence the immune functions of leukocytes largely by regulating their mobilization and redistribution between the bone marrow and the peripheral tissues or circulation, with a similar outcome featuring relocalization of leukocytes back in the bone marrow [17–20]. These findings in fasting invention affecting the immune function and relocalization of leukocytes to the bone marrow have been primarily reported with mice undergoing fasting for several hours. Roughly, several hours of fasting for mice is comparable to several days of fasting for humans, taking into consideration the huge discrepancy in lifespan (2 years vs. 80 years) and maximum fasting days (3 days vs. 30 days) between mice and humans. This brings in an unanswered question on how the human immune system responds to the increased risk from invasion by infectious pathogens during the period of fasting. Our study suggests that the increased risk of infectious diseases during fasting may be remedied by activation of the immune function of red blood cells triggered by fasting. Therefore, red blood cells have the capacity to likely function as sentry when leukocytes are in shortage in circulation during starvation stress.

Red blood cells are implicated in modulating immune function through complement receptors on their membranes [8–13, 30], cytokines expressed in red blood cells [31, 32], or through controlling the function of other cells, including T lymphocytes, macrophages, dendritic cells, neutrophils, and eosinophils [24]. Omics studies provide new insight into the immune function of red blood cells. A recent finding by single-cell transcriptomic analysis revealed the immunomodulatory capacity of red blood cells throughout human development, suggesting a long-lasting immune function of immature erythrocytes in humans [16]. Nevertheless, single-cell RNA sequencing is a powerful way to resolve the function of nucleated cells, not enucleated cells such as mature red blood cells. Our recent study with proteomics and biochemical tools shows that in humans, short-term intensive fasting remodels the innate immunity of neutrophils disclosed on their expression profiles [21] and reduces thrombosis risk without compromising the hemostasis capacity of platelets [33].

Fasting has been reported to coordinately affect the proportion of polyunsaturated versus saturated and monounsaturated fatty acids at the erythrocyte membrane, leading to protection of chemotherapy toxicity in mice and humans [34], suggesting that fasting may be used to influence erythrocyte membrane dynamics. In our present study, the proteome of red blood cells shows that STIF leads to extensive enrichment in their immune function (Fig. 1), and among the top ten pathways in red blood cells activated by STIF, the majority are pertinent to immune responses to pathogens ranging from viruses and bacteria to parasites (Fig. 2). Based on our current understanding of the mechanism by which red blood cells are implicated in immune protection [8–13, 30] [31, 32] [24], we infer that STIF may enhance red blood cells to work with other components of the immune system, such as macrophages, to fight against various pathogens from invasion.

Recently, *SARS-CoV-2* spikes or C3 have been detected on the red blood cells of COVID-19 patients [25, 35–37], and an acquired decrease in CR1 and increased deposits of C4 fragments on red blood cells among COVID-19 patients have also been found [26], suggesting that the handling and clearance of immune complexes or complement fragment-coated cell debris may play an important role in the pathophysiology of COVID-19. These reports indicate the participation of complement regulatory proteins and indicate that red blood cells are important in the immune pathophysiology of COVID-19 patients [26]. Our proteomic analysis shows that STIF-activated immune responses are mostly complement activation, which leads to an augmentation in both alternative and classical pathways in the coronavirus disease (COVID-19) pathway (Fig. 3). Our finding is in agreement with recent documentation that the red blood cell membrane may respond to *SARS-CoV-2* infection [25, 26]. Therefore, oxygen-carrying red blood cells may capture pathogens such as *SARS-CoV-2* via complement receptors expressed on their membrane, and fasting enhances the activation of complements, leading to a stronger capacity to fight against pathogen invasion.

Encouragingly, biochemical assays suggested that the activation of complements recruited by their receptors to red blood cell membranes triggered by STIF did not alter the oxygen delivery capacity or survival of red blood cells (Figs. 4 and 5). It thus appears that there are no detectable side effects on red blood cells after STIF. However, the major limitation of this study is that no comparison was made between the impact of STIF on the functional changes in red blood cells from healthy subjects and patients infected by pathogens such as *SARS-CoV-2*. Therefore, our contention needs to be further tested in future studies, particularly examining the long-term effect of such an intervention on the immune function of red blood cells.

**Fig. 6:**
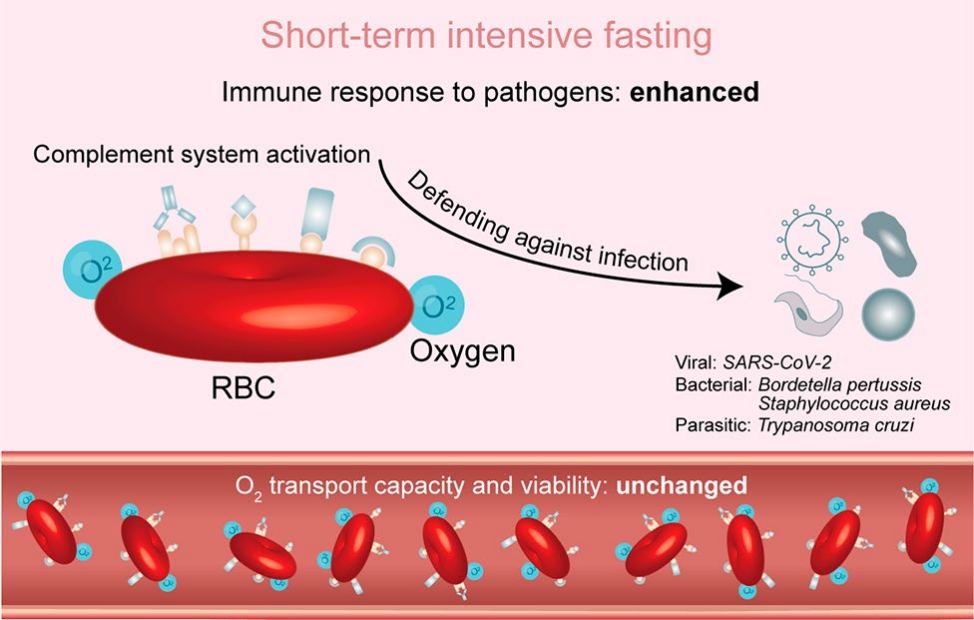
Cartoon illustrating the argument of the immune function of red blood cells by STIF. STIF triggers the activation of the complement system via complement receptors on the membrane of red blood cells to enhance the immune response to various pathogens, particularly *SARS-CoV-2*. When empowered with upregulated immune function by STIF, red blood cells maintain their oxygen transport capacity and viability

In conclusion, proteomic, biochemical and flow cytometric analyses suggest that STIF enhances the immune function of red blood cells via complement activation but not at the cost of oxygen transport and viability. The impact of STIF on the immune function of red blood cells is summarized in Fig. 6. We speculated that the immune function of red blood cells enhanced by STIF may be extremely important in combating pathogen invasion due to their dominance in cell number among all types of cells in circulation. Therefore, this finding is important for our understanding of how “nonimmune cells”, in particular red blood cells, respond to remedy the shortage of immune cells in circulation under fasting stress.

## Methods

### Study design

The study was conducted with approval from the Institutional Ethics Review Board at Soochow University (Approval No. ECSU-2,019,000,153) and the Chinese Clinical Trial Register, an official review board for clinical trials (Registration No. ChiCTR1900027451), affiliated with The Ministry of Public Health of China (http://www.chictr.org.cn/index.aspx). Participants were enrolled after giving their written informed consent. Thirty-one participants aged 27 to 67 years were enrolled from several provinces of China. Exclusion criteria and fasting programs as well as refeeding protocols were described previously [33]. Briefly, participants were starved of only water for 6 days. Then, they were gradually refed for 6 days. During refeeding days, they ate soup made of millet and rice for 2 days, gradually increased the intake of oil and salt, and gradually increased the types of food consumed by noodles, eggs, and vegetables. After 6 days of refeeding, the diet was basically restored to the diet before STIF. Blood samples were collected at 7:00 to 9:00 am on the testing days indicated in the figures.

### Blood routine analysis

Blood was collected in a sodium citrate anticoagulant tube. We performed routine five-class blood analysis and analyzed the MCV and RDW of each group of humans.

#### Morphological detection of RBCs

RBCs were obtained from PB by centrifugation at 3000 rpm/min for 10 min. RBCs were transferred to glass slides by centrifugation at 400 rpm/min for 5 min. A Wright-Giemsa stain kit (D010, Nanjing JianCheng Technology) was used to detect the morphology of the RBCs according to the manufacturer’s instructions.

### 2,3-DPG assay

We collected the PB of each group of humans. Red blood cells were obtained by centrifugation at 3000 rpm/min for 10 min. The concentration of 2,3-DPG was determined using a human ELISA Kit (A099975, Shanghai Fusheng Industrial/Affandi) on a Labsystems Multiskan MS 352 Microplate Reader (Thermo Fisher Scientific) according to the manufacturer’s instructions.

### PfHb assay

f-Hb levels in plasma were assayed by enzyme-linked immunosorbent assay (ELISA) using a human f-hb ELISA Kit (A011547, Shanghai Fusheng Industrial/Affandi) according to the manufacturer’s instructions.

### Proteome sequencing in human red blood cells

#### Protein extraction and quality control

An appropriate amount of sample was transferred to a 1.5 mL centrifuge tube, and an appropriate amount of protein lysis buffer containing SDS (sodium dodecyl sulfate) and a final concentration of protease inhibitor cocktail with EDTA was added. Ultrasound in ice bath, centrifuge at 25, 000 g 4 °C for 15 min, take the supernatant and add DTT (dithiothreitol) with a final concentration of 10 mM, and place in a water bath at 37 °C for 30 min. Add IAM (Iodoacetamide) with a final concentration of 55 mM, and place in a dark room for 45 min. Add 5 times the volume of precooled acetone, place in -20 °C refrigerator for 2 h, centrifuge at 25, 000 g 4 °C for 15 min and discard the supernatant. The precipitate was air-dried, an appropriate amount of SDS-free protein lysis solution was added, and an automatic grinder was used to promote protein dissolution. Centrifuge at 25,000 g and 4 °C for 15 min, and take the supernatant, which is the protein solution. Standard proteins (0.2 µg/µL BSA) (0, 2, 4, 6, 8, 10, 12, 14, 16, and 18 µL) were sequentially added to 96-well microtiter plates A1 to A10, followed by the addition of pure water (20, 18, 16, 14, 12, 10, 8, 6, 4, and 2 µL), and then 180 µL of Coomassie Brilliant Blue G-250 Quantitative Working Solution was added to each well. The OD595 was measured with a microplate reader, and a linear standard curve was drawn based on the OD595 and protein concentration. The protein solution was diluted several times for testing, 180 µL of the quantitative working solution was added to 20 µL of the protein solution, and the OD595 was read. The sample protein concentration was calculated from the standard curve and sample OD595. Each 10 µg of protein solution was mixed with an appropriate amount of loading buffer, heated at 95 °C for 5 min, and centrifuged at 25,000 × g for 5 min, and the supernatant was loaded into a well of a 12% SDS polyacrylamide gel. After electrophoresis, Coomassie blue staining was carried out for 2 h, after which an appropriate amount of decolorizing solution (40% ethanol 10% acetic acid) was added to the shaker to decolorize 3 to 5 times for 30 min each time. One hundred micrograms of protein solution per sample was diluted with 50 mM NH_4_HCO_3_ in 4 volumes. Two micrograms of trypsin enzyme was added at a protein:enzyme ratio of 40:1 and digested for 4 h at 37 °C. Enzymatic peptides were desalted using a Strata X column and vacuumed to dryness.

#### High pH RP separation

Equal amounts of peptides were extracted from all samples to mix, and the mixture was diluted with mobile phase A (5% ACN pH 9.8) and injected. A Shimadzu LC-20AB HPLC system coupled with a Gemini high pH C18 column (5 μm, 4.6 × 250 mm) was used. The sample was subjected to the column and then eluted at a flow rate of 1 mL/min by gradient: 5% mobile phase B (95% ACN, pH 9.8) for 10 min, 5–35% mobile phase B for 40 min, 35–95% mobile phase B for 1 min, flow phase B lasted 3 min and 5% mobile phase B equilibrated for 10 min. The elution peak was monitored at a wavelength of 214 nm, and the components were collected every minute. Components were combined into a total of 10 fractions, which were then freeze-dried.

#### DIA quantification (Nano-LC–MS/MS)

The dried peptide samples were reconstituted with mobile phase A (2% ACN, 0.1% FA) and centrifuged at 20,000 × g for 10 min, and the supernatant was taken for injection. Separation was carried out by a Thermo UltiMate 3000 UHPLC liquid chromatograph. The sample was first enriched in the trap column and desalted and then entered a tandem self-packed C18 column (150 μm internal diameter, 1.8 μm column size, 35 cm column length) and separated at a flow rate of 500 nL/min by the following effective gradient: 0 ~ 5 min, 5% mobile phase B (98% ACN, 0.1% FA); 5 ~ 90 min, mobile phase B linearly increased from 5 to 25%; 90 ~ 100 min, mobile phase B rose from 25 to 35%; 100 ~ 108 min, mobile phase B rose from 35 to 80%; 108 ~ 113 min, 80% mobile phase B; 113.5 ~ 120 min, 5% mobile phase B. The nanoliter liquid phase separation end was directly connected to the mass spectrometer as the following settings. The peptides separated by liquid phase chromatography were ionized by a nanoESI source and then passed to a tandem mass spectrometer Oritrap Exploris 480 (Thermo Fisher Scientific, San Jose, CA) for DDA (Data were ionized Acquisition) detection mode. The main parameters were set as follows: the ion source voltage was set to 1.9 kV, the MS1 mass spectrometer scanning range was 400 ~ 1,250 m/z, the resolution was set to 120,000, the maximal injection time (MIT) was 90 ms, and 400 ~ 1,250 m/z was equally divided into 50 continuous window MS/MS scans. MS/MS collision type HCD, collision energy NCE 30; MIT was auto mode. Fragment ions were scanned in Orbitrap at an MS/MS resolution of 30,000. AGC was MS 300%, MS/MS 1000%. The DIA data were analyzed using the iRT peptides for retention time calibration. Then, based on the target-decoy model applicable to SWATH-MS, false positive control was performed with an FDR of 1%, therefore obtaining significant quantitative results.

#### MSstats differential analysis

MSstats [38] is used for statistical evaluation of significant differences in proteins or peptides. The data were preprocessed according to the predefined comparison group, and then a significance test was performed based on the linear mixed model. Thereafter, differential protein identification was performed based on a |log_2_(fold change)|≥log_2_(1.5) and a *P* value < 0.05 as the criteria for statistically significant differences. We drew a volcano plot and heatmap using the python library bioinfokit[39].

#### Overrepresentation analysis

Overrepresentation analysis was performed on DEPs identified from differential analysis using clusterProfiler [22] on GO and KEGG. The criterion for identifying significantly overrepresented terms was filtered by *P* value < 0.05. Venn diagrams were plotted using the Python library matplotlib or custom Venn scripts.

#### Gene set enrichment analysis

We conducted GSEA on preranked gene lists with log_2_(fold change) as the ranking metric using clusterProfiler. We used GO terms or KEGG pathways as gene sets.

### Data presentation and statistical analysis

Statistical data were analyzed using SPSS version 22.0. In general, data with more than two groups were analyzed using one-way analysis of variance (ANOVA) with multiple comparisons (using Dunnett’s test with fasting day 0 as the control group). *P* values from one-way ANOVA are presented as #. *P* values from multiple comparisons were corrected using the Benjamini & Hochberg method and reported using asterisks (Adjusted *P* value, ns ≥ 0.05, * <0.05, **<0.01, ***<0.005). Plots were generated using GraphPad Prism 8, and data are presented as the mean ± standard error of the mean (SEM).

### Data Availability

Proteome profiles are being uploaded to the ProteomeXchange platform. Any additional data that support the findings of this study are available from the corresponding authors upon request: jrwang@suda.edu.cn, or nyuan@suda.edu.cn.

### Electronic supplementary material

Below is the link to the electronic supplementary material.

Supplementary Material 1
