# Supplementary material for: Short-term intensive fasting enhances the immune function of red blood cells in humans
Source: Immun Ageing. 2023 Aug 30;20:44. doi: 10.1186/s12979-023-00359-3 (PMC10469874; doi:10.1186/s12979-023-00359-3)
Supplement: Supplementary file 1 — Supplementary Material 1 [file 12979_2023_359_MOESM1_ESM.pdf]

Supplemental Information

Short-term intensive fasting enhances the immune function of red blood cells in humans

Table S1. Information of STIF participants

Participant numbers	Gender	Age	Ethnicity	Proteome of RBC	SBP (mmHg)	DBP (mmHg)	Heart rate (Beats/min)	BBT (℃)
1	Female	67	Han, Chinese	-	162	88	63	36.7
2	Male	61	Han, Chinese	-	132	63	57	36.4
3	Male	60	Han, Chinese	-	126	72	60	36.5
4	Female	58	Han, Chinese	-	129	82	73	37.1
5	Female	57	Han, Chinese	-	129	77	72	36.4
6	Female	51	Han, Chinese	-	150	91	75	36.9
7	Male	50	Han, Chinese	✔	141	94	67	36.3
8	Female	49	Han, Chinese	✔	122	82	75	36.7
9	Male	48	Han, Chinese	-	117	71	67	36.4
10	Female	47	Han, Chinese	-	94	57	59	36.2
11	Male	46	Han, Chinese	✔	118	74	72	36.4
12	Female	44	Han, Chinese	-	118	68	73	36.7
13	Female	41	Han, Chinese	-	114	66	72	36.6
14	Female	41	Han, Chinese	-	90	61	64	36.3
15	Male	41	Han, Chinese	✔	116	72	71	36.4
16	Female	40	Han, Chinese	-	114	65	79	37.0
17	Female	40	Han, Chinese	-	105	58	63	36.4
18	Female	40	Han, Chinese	-	113	75	76	36.8
19	Male	39	Han, Chinese	-	126	67	50	36.7
20	Male	37	Han, Chinese	-	109	68	66	37.1
21	Female	37	Han, Chinese	-	143	107	94	36.3
22	Female	36	Han, Chinese	-	105	51	69	36.4
23	Female	35	Han, Chinese	✔	121	69	72	36.7
24	Female	34	Han, Chinese	-	102	59	86	36.7
25	Male	34	Han, Chinese	-	139	86	83	36.8
26	Female	34	Han, Chinese	✔	107	61	72	36.7
27	Male	32	Han, Chinese	-	136	88	90	36.4
28	Female	32	Han, Chinese	-	113	67	78	36.8
29	Male	31	Han, Chinese	-	132	81	74	36.3
30	Female	31	Han, Chinese	-	94	54	85	36.9
31	Female	25	Han, Chinese	-	114	64	73	37.4

STIF (short-term intensive fasting)
RBC (red blood cell)
SBP (systolic pressure)
DBP (diastolic pressure)
BBT (basal body temperature)
FBG (fasting blood-glucose)

In this study, Samples of red blood cells of the 31 participants were all included in biochemical and flow cytometric analysis. To control cost, we randomly selected blood samples of only 6 participants (3 males and 3 females) from the entire STIF cohort for proteomic sequencing. To systemically evaluate and classify samples, we collected 5 metrics, including percentage of $GPA^{+}CD35^{+}$ in peripheral blood cells, MPV, RDW, PfHb and 2,3-DPG and ran unsupervised clustering on these samples using K-Means (plotted in two-dimensional principal component space). The 6 samples, which were selected at random and used for sequencing, is annotated in the scatter plot with yellow outlines and numeral labels (Figure S1). Samples are indexed with the same numeric ordering as Table S1. As illustrated in the following scatter plot, the six samples are clustered in cluster 0 and distributes uniformly in the PCA space, indicating ideal representations for our cohort.

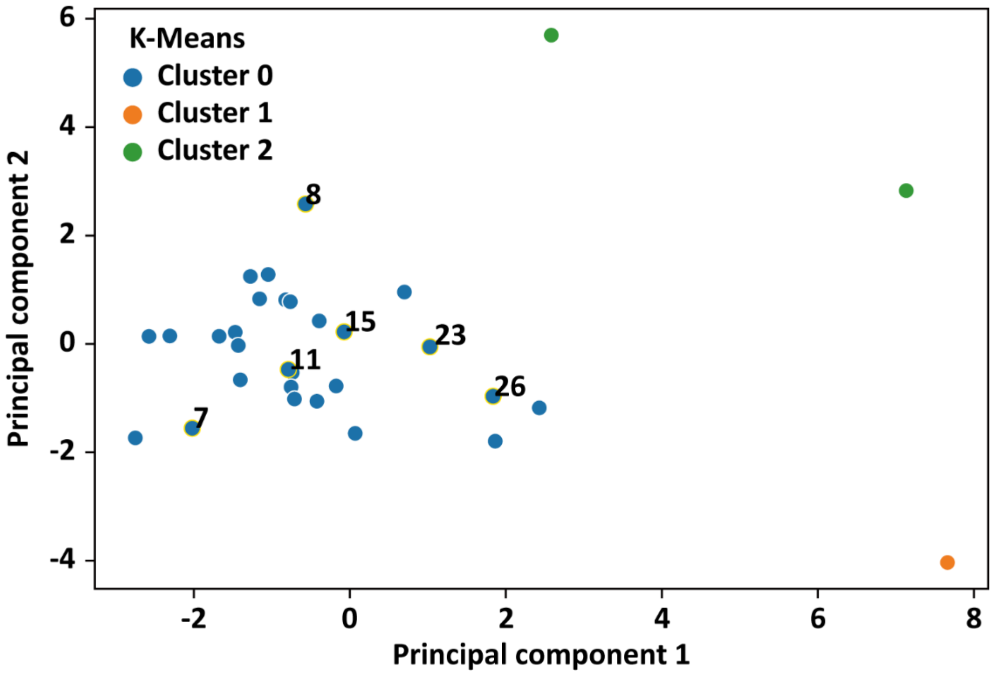

Figure S1. Scatter plot showing sample selection for proteomic sequencing. **S**amples 7, 8, 11, 15, 23 and 26 were chosen randomly.
